# Winners and runners-up alike?—a comparison between awardees and special mention recipients of the most reputable science award in Colombia via a composite citation indicator

**DOI:** 10.1057/s41599-022-01241-1

**Published:** 2022-06-28

**Authors:** Julián D. Cortés, Daniel A. Andrade

**Affiliations:** 1 School of Management and Business, Universidad del Rosario, Bogotá, Colombia; 2 Fudan Development Institute, Fudan University, Shanghai, China; 3 School of Business, Woxsen University, Hyderabad, India; 4 Independent, Bogotá, Colombia

**Keywords:** Science, technology and society, Education

## Abstract

The research agenda on global academic elites (e.g., those awarded the Nobel Prize) has overlooked academic awards and elites from developing countries and the public symbolic recognition of scientific elites by research awards. In this study, we examine the bibliometric features of individual researcher profiles of those participants who received a special mention in Colombia’s most prestigious prize in the sciences: the Alejandro Ángel Escobar Prize (AAEP). First, we chart the citation per article trend of Colombia’s most prolific researchers before and after receiving the special mention and the AAEP. We then compare the special mention group with those awarded the AAEP, using a composite citation indicator of six scientific impact and productivity indices to estimate (1) bulk impact (number of citations and *h* index) and (2) authorship order adjusted impact (Schreiber *hm* index; total citations for articles of which the scientist is the single author; total citations for articles of which the scientist is the single or first author; and total citations for articles of which the scientist is the single, first, or last author). Results show that there is no overall *halo effect* in citation per article after receiving the special mention or the AAEP. Such recognition comes after an academically productive career marked by multiple citations per article peaks. There is no clear-cut division between the composite citation indicator of those awarded a special mention and those awarded the AAEP. Findings place the profile of local authors in an adjusted and inclusive framework that takes full cognisance of the scientific elites in developing countries.

## Introduction

A former Colombian soccer coach—who also is a former dentist—coined a saying that briefly turned him into an amateur philosopher: *perder es ganar un poco* (losing is winning a little bit). Among those who *win* the prestige of being part of the global scientific elite are the Nobel Prize laureates and winners of other international awards such as the Fields Medal or Turing Award. Disentangling the research output, impact and structure of the global scientific elite is fertile ground for researchers. For example, a Boolean search on Scopus’s bibliographic database with the keyword “Nobel Prize” limited to 14 core journals on informetrics and research evaluation (Waltman, 2016) such as *Journal of Informetrics* or *Scientometrics* returned 75 results (Scopus, 2020a). However, two factors have hitherto been excluded from this research agenda: scientific awards in developing countries; and those participants who were nominated but failed to get the *laurels*, i.e., those who *won—a little bit*. The latter factor is difficult to assess, not least because Nobel Prize nominees cannot be revealed until 50 years after the award has been granted (The Nobel Prize Foundation, 2021).

Such inquiries are pressing in the research evaluation context of developing countries where output path-dependent trajectories, research focus, and motivation diverge from those of the Global North (Confraria and Godinho, 2015; Confraria et al., 2017; Klavans and Boyack, 2017; Cortés-Sánchez, 2019, 2020; Cortés, 2021a, 2021b, 2022; Cortés et al., 2021a, 2021b). Consider the case of Colombia, ranked 5th in Latin America with 123,000+ citable documents and a *national* h index of 333 (Hirsch, 2005) (1996–2020) (SCImago, 2021). Despite occupying a regional top-five position, such accomplishments fade when viewed through the lens of global scientific elite standards. Only two Colombian individuals have been lauded with Nobel Prizes: Gabriel García Márquez in literature, and Juan Manuel Santos for peace. There have been none in the sciences (The Nobel Prize Foundation, 2021). Among Clarivate’s Highly Cited Researchers, Colombia has only one researcher: Olga Sarmiento, Universidad de Los Andes (Clarivate Analytics, 2020). Nor has a Colombian ever been awarded either a Fields Medal or the Turing Award—equivalents to Nobel Prizes in mathematics and computing, respectively. Are we to deduce from this that a Colombian scientific elite is non-existent? On the contrary, Colombia has its own Nobel Prize equivalent: the *Alejandro Ángel Escobar Foundation National Prize* (AAEP) (Dinero, 2000; Faciolince, 2007).

Alejandro Ángel Escobar was born into a wealthy family. His father, Alejandro Ángel Londoño, moved with his family to New York in 1906, where Escobar finished elementary and high school. He then went on to study Economics at the University of Cambridge. In 1927, he returned to Colombia, where he held executive roles in the private and public sectors. His cosmopolitan mindset and firm belief in the importance of science for society’s progress motivated him to investigate the functioning of both the Nobel and Rockefeller foundations with a view to supporting and incentivising Colombian science and solidarity. Unfortunately, Escobar’s life was cut short at the age of 50 and it was left to his wife, María Restrepo de Ángel, to realise his husband’s vision. In 1954, she created the *Alejandro Ángel Escobar Foundation* (AAEF) (Fundación Alejandro Ángel Escobar; Faciolince, 2007) and remained its President for almost 40 years.

As stated in Escobar’s will, the Foundation’s prizes have the following aim: ‘*The prizes are to be assigned to truly outstanding work, which merits the seal of excellence, if not absolutely, then at least within the country’s cultural horizons. It is not my wish that the least bad be awarded, but the really good*.’ (Fundación Alejando Ángel Escobar, 1953). Since 1955 the AAEF has awarded three categories in the sciences: (1) *Physics and Natural Sciences*; (2) *Social Sciences and Humanities*; and (3) *Environmental Sciences and Sustainable Development*. There is also a *solidarity* category that awards social development programs in Colombia, which was not discussed here since is not related with research in the sciences. This particular prize consists of a silver medal, a diploma, and a sum of cash awarded to the leading researcher/representative/or coordinator of the work. Each year, Colombian authors—irrespective of institutional affiliation—are invited to submit their research for assessment by the Foundation’s committee. The leading researcher/representative/or coordinator role has to be indicated by the authors if the work is not sole-authored. Nevertheless, each author must endorse the submission in the case of multiple authors. In any case, the representative/leading researcher/coordinator should be of Colombian extraction. The AAEF accepts master’s or PhD theses, technical reports, and research articles or books. In addition to the award itself, there are also *special mentions* for outstanding work in each category when applicable.

Requirements of the AAEP review process are (Fundación Alejandro Ángel Escobar, 2020): a researcher can not participate with an application submitted in a previous year or submit several applications to the same category in the same year; the Foundation’s board of directors will select the Foundation committee responsible for evaluating the submissions; selection of the Foundation committee members is based on their scholarly, professional, and human accomplishments; the Foundation committee will assess the submissions under impartial Foundation supervision; and the Foundation committee is free to assign—or not—the prize(s) and special mention(s) based on the merits of the submission. The Foundation committee can assign a maximum of two special mentions.

One of the most important differences between the AAEP and other scientific elite awards, such as the Nobel Prize or the Fields Medal, is that the researchers submit their work to be considered a nominee for the AAEP—not the case for the last two prizes. Yet, despite these criteria of self-nomination and openness to both junior to senior researchers, thirty-one per cent of the AAEP awardees have completed an academic degree in the US and 28% a PhD in reputable universities such as Harvard, MIT, Yale, or Wisconsin-Madison (Cortés and Andrade, 2022). Further, 27.6% have completed a degree in an elite public or private institutions in Colombia, such as Nacional, Antioquia, Valle, or Los Andes (Cortés and Andrade, 2022). It supports the findings that top-tier institutions generate the most output and impact in several fields and affiliations with global and local scientific elites (Morgan et al., 2018). In other words, AAEP awardees display aspects of academic top-tier academic pedigree despite its self-nomination mechanism.

In addition, Nobel Prize committees assess nomination forms that are sent to thousands of members of academies, universities, parliamentary assemblies, and previous Nobel laureates (The Nobel Prize Foundation, 2021). Then, specially appointed experts advise the Nobel Committees on the candidates’ extended work (The﻿ Nobel Prize Foundation, 2021). Therefore, it could be reasonably assumed that Nobel Prizes are awarded for a devoted researcher’s total sum of work. However, sciences Nobel Prize achievements are in the form of scientific papers (Zhou et al., 2014). For instance, Georg Bednorz and Alex Müller received the Nobel Prize the next year of publishing their article on high-temperature superconductors (Müller and Bednorz, 1987). Consequently, studies on scientific elites are focused on landmark research papers (Zhou et al., 2014) or the complete research record of awardees (Li et al., 2019a, 2020; Jin et al., 2021). This study lies in the latter stream.

In sum, by comparing awarded researchers with those who received a *special mention*, we evaluated the research impact of those who *won a little bit* relative to those who *won*. The former group denotes the Colombian scientific semi-elite (sCSE), and the latter the Colombian scientific elite (CSE). Global scientific elites push science’s boundaries and hold significant influence and power over university departments/institutes, scientific societies and award and funding bodies (Ma and Uzzi, 2018). However, such comparative views in scientific elites have to be grounded in the context of middle-low-income regions. Colombia, in that line, has the lowest share of tertiary education for adults (25–64) among OECD countries, with 23% (OECD, 2019). In consequence, in this study, a researcher holding a graduate degree in a local/global elite institution and being awarded the most reputable science award in the country—or its special mention—despite the gap in influencing a field on a global scale, will be addressed as a member of such a (semi)elite.

This study aims to examine the bibliometric features of individual researcher profiles of the sCSE and to compare the research impact of that group with that of the CSE via a composite citation indicator. The research questions (RQs) that guide this study are as follows:

- RQ_1_: What are the overall bibliometric features of the sCSE?
- RQ_2_: Are the sCSE and CSE two completely distinctive and fractionalised groups in terms of scientific impact?

Contributions to these RQs provide a nuanced perspective of scientific (semi-)elites in developing countries, such as a research impact view between both groups adjusted by overall impact, number of coauthors, and their research leading role (Ioannidis et al., 2016). This information will enable research evaluation organisations and individuals in developing countries to make an informed decision as to whether local scientific semi-elites or even non-elite researchers are significantly different (Li et al., 2019b). On a minor note, the research output, impact, and structure of the CSE its been discussed elsewhere (Cortés and Andrade, 2022). Following this introduction, section “Research background” presents the research background, followed by section “Methodology and materials” with methodology and materials. Results are presented in section “Results” and discussed in section “Discussion”. The conclusion outlines the main findings, limitations, and future agenda in section “Conclusion”.

## Research background

The work of Harriet Zuckerman, in which qualitative methods were used to chart the career trajectories of Nobel laureates, is regularly cited as the seminal in-depth study of the scientific elites in the US (i.e., Nobel laureates) (Zuckerman, 1977). A few years earlier, however, Eugene Garfield—discussing the use of citation indexing for studying the sciences—discovered that among the fifty most cited researchers for 1967 were two Nobel laureates awarded in 1969: Derek H. R. Barton and Murray Gell-Mann. ‘[N]o small achievement,’ he wrote (Garfield, 1970), and a discovery that would open a rich vein of enquiry.

Garfield found that Nobel laureates of the 1960s were cited ~30 times as often as the average researcher and were authors of multiple *citation classics* (i.e., documents cited over 300 times); and that the use of a simple technique to identify high rankings by citation and citation/paper was sufficient to corroborate and forecast laureates-in-waiting (Garfield, 1986; Garfield and Welljams-Dorof, 1992)—a claim later disputed by Gingras and Wallace (2010). In a study contemporaneous with Garfield’s, Stephan and Levin (1993) analysed the relationship between age and productivity in Nobel laureates from 1901 to 1992. They found that age distribution differs by field. For instance, the mean age in chemistry was 37.8, physics: 36, and medicine/physiology: 39. Youth is not, therefore, a determinant of Nobel-type creativity, whereas the latter was found to decrease in mid-life—a subject later revisited by Abramo et al. (2016), who estimated the average Nobel laureate’s age at 44.1 ± 9.7.

The first decade of the second millennium brought more substantial and diversified research on scientific elites driven by the increasing digitalisation of bibliographic records and computing capacity for large-scale datasets (Li et al., 2020). From 2010 on, the scientific elite category was enlarged to encompass other highly reputable awards—in developing countries, however, scientific awards have been sidelined. Research topics on Nobel laureates have included: regularities and tendencies (e.g., the time interval between discovery and recognition, document characteristics, or pre-Nobel resistance within the scientific community to the idea of such a prize) (Karazija and Momkauskaite, 2004; Campanario, 2009; Ma et al., 2012; Li et al., 2019b; Bjørk, 2020; Sebastian and Chen, 2021); productivity, collaboration, impact-citation, and research field structures (Kademani et al., 2005; Bjork et al., 2014; Wagner et al., 2015; Chan et al., 2016; Ioannidis et al., 2020; Kosmulski, 2020); gender bias (Lunnemann et al., 2019); and the appearance on the Nobel podium of scientists from the developing world (Heinze et al., 2019)

Related research on scientific elites, focused on identifying elite scientists outside the US (i.e., Canada) (Larivière et al., 2010), map other renowned international awards (e.g., The Royal Medal; Max Planck; Darwin Medal), their relationships, genealogical-authorship networks, and how it can predict future breakthroughs in science (Zheng and Liu, 2015; Ma and Uzzi, 2018). More recent research has adopted the Zuckerman approach but applied it specifically to awards other than the Nobel, such as the Turing Award and the Fields Medal (Chang and Fu, 2021; Jin et al., 2021).

Despite the substantial body of evidence on the global scientific elite, two factors have been excluded from this research agenda: scientific awards in developing countries; and those participants who were nominated but failed to get the award. The following section will present the methodology and materials sourced and implemented to address this research gap.

## Methodology and materials

### Data

**Fig. 1:**
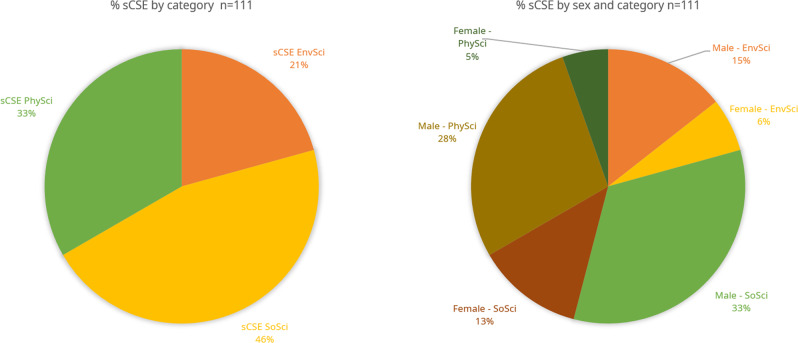
Percentage of sCSE by category (left); and percentage of sCSE by sex and category (right). Source: Fundación Alejandro Ángel Escobar (2007, 2020). Note: EnvSci: *Environmental Sciences and Sustainable Development*; SoSci: *Social Sciences and Humanities*; PhySci: *Physics and Natural Sciences*.

The list of both the sCSE and CSE was sourced from the AAEF website (2000–2020) and a book published by the AAEF (1990–1999) commemorating its half-century in existence (Fundación Alejandro Ángel Escobar, 2007). The sample was limited to the period 1990–2020, given that Colombia’s international publishing profile dates from the early 1990s (Villaveces and Forero-Pineda, 2007). Figure 1 presents the sCSE sample of 111 awardees as % of the sCSE by category and by % of the sCSE by sex and category. Female researchers have a total ~24% participation among the awardees, with the highest participation in the social sciences and humanities category. By contrast, male researchers have a total ~75% participation among the awardees, with the highest participation in physics and natural sciences category.

According to the Colombian Ministry of Science, Technology, and Innovation platform for researchers’ curriculum vitae (CvLAC), most of the sCSE have under/graduate studies in Colombia: ~32%, and the US: ~25%, followed equally by Spain, France, and the UK (~6%) (Minciencias, 2021). The most frequent local university affiliations were: Universidad Nacional (public) and Universidad de Los Andes (private). Among the US universities, the most frequent were Harvard University (private) and the University of Wisconsin (public).

Studies on the scientific elite have covered both the most influential work of the researcher (i.e., the work for which he/she was awarded) (e.g., Sebastian and Chen, 2021) or the complete set of publications during his/her career (e.g., Zuckerman, 1977; Li et al., 2020; Stephan and Levin, 1993). We adopted the latter approach since—with the current availability of bibliographic data and complete profile of researchers—it is crucial to extend our understanding from the prize-winning work to the broader context of the researchers’ entire career (Li et al., 2020).

There are multiple bibliographic search engines and databases from which scientometric researchers source bibliographic data (Gusenbauer, 2019). A recent assessment found that the one with the most extensive coverage is Google Scholar, followed by Microsoft Academic, Scopus, Dimensions, and WoS (Web of Science) (Martín-Martín et al., 2021). However, each of them has particular coverage and accuracy features. For instance, WoS and Scopus are selective since the scholarly communications cover specific standards (e.g., excluding journals with predatory features), while the others aim to be comprehensive (e.g., data curation is performed using artificial intelligence with a minimum of interference from humans) (Waltman and Larivière, 2020). Search engines such as Google Scholar show limitations in search interface and query optimisation, exporting many cited references, and a visual search builder (Boeker et al., 2013). Furthermore, since Google Scholar records are added externally, researchers shall revise their profile in search of erroneous additions or duplications that may lead to abrupt inflations of their citation profile (López-Cózar et al., 2014; Teixeira da Silva JA, 2018).

We selected Scopus for this study. Scopus has a more comprehensive journal coverage and broader social science inclusion than WoS (Mongeon and Paul-Hus, 2016; Baas et al., 2020; Waltman and Larivière, 2020). Also, despite being an Anglo-American/Western-oriented database, the international coverage authorship in Scopus from higher-income countries such as the US, the UK, or Germany showed a decrease since 2000, while developing regions have increased their participation significantly (e.g., China and India) (Thelwall and Sud, 2022).

We search for each sCSE profile in Scopus by complete name. We cross-checked each researcher’s past/current affiliation to exclude homonymous researchers. We also limited the articles analysed to the period 1996–2020 in view of Scopus’s indexing fidelity (Baas et al., 2020). Researchers with just one indexed publication were excluded. We found 35 sCSE research profiles in Scopus with the correspondent bibliographic record, constituting 31% of the sCSE complete list of 111 researchers. For researchers with multiple *special mentions*, only the initial one was considered for chronological-related analysis.

We excluded documents with 10+ researchers. In the field of Big Science (De Solla Price D, 1963), the involvement of multiple researchers, institutions, and countries is a common factor—particularly in high-energy physics. However, while multi-authored (+10 researchers) and hyper-authored (+100 researchers) articles are on the rise, they are still the exception: 95% of the global output publication in the Web of Science (WoS) has ten or fewer researchers (Adams et al., 2019). Either way, such articles deserve a full analysis, but it is one that lies outside the scope of this study since it significantly skews output and impact, particularly in PhySci. A total of 399 articles with 10+ researchers were excluded from our analysis due to the difficulty of assessing researcher contribution, accountability, and credit (Cronin, 2001; Thelwall and Fairclough, 2020). Most of these articles related to medicine: 35%; biochemistry, genetics, and molecular biology: 26%; and agricultural and biological sciences: 12%.

**Fig. 2:**
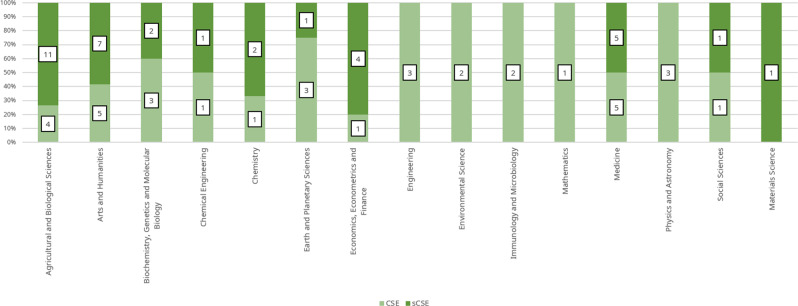
sCSE and CSE sample by ASJC subject. Source: (Fundación Alejandro Ángel Escobar, 2020; Scopus, 2021; Fundación Alejandro Ángel Escobar 2007; Aria and Cuccurullo 2017; Scopus 2020a; Minciencias 2021).

We also sourced and matched a sample of the CSE bibliographic profile from Scopus to address RQ_2_ as follows:

- We re-classified both the sCSE and CSE according to the most frequent subject of the journals in which each researcher has published. For instance, special mention researchers in the EnvSci category ranged from biologists to economists. This produced a refined profile according to each researcher’s output. The re-classification was conducted as follows:
  ○ We used the Scopus research profile of 41 CSE 1990–2020 sourced previously in another study (Cortés﻿ and Andrade, 2022).
  ○ The Scopus profile sample of CSE (*n* = 41) was larger than that of sCSE (*n* = 35). Therefore, we chose 35 CSE researchers at random, matching the sCSE sample, for a final sample of 70 profiles.
  ○ We then cross-checked each journal’s printed-ISSN in which the sCSE and CSE have published, together with its correspondent All Science Journal Classification (ASJC) (Scopus, 2021) subject area.
  ○ If a given journal belonged to more than one ASJC subject area, it was randomly assigned.
  ○ After identifying the most frequent ASJC subject area, each researcher was then assigned to the respective area. Figure 2 displays both the sCSE and CSE samples by ASJC subject.
- The complete sample of 70 researchers is similar to other studies on academic elites (e.g., *Derek John de Solla Price Medal:* 29*; Turing Award*: 72) (Hou et al., 2021; Jin et al., 2021). Most of the sCSE work in the fields of agricultural and biological sciences, followed by arts and humanities, and medicine, whereas the CSE work mostly in arts and humanities, and medicine, followed by agricultural and biological sciences, and biochemistry, genetics and molecular biology (Fig. 2).

**Fig. 3:**
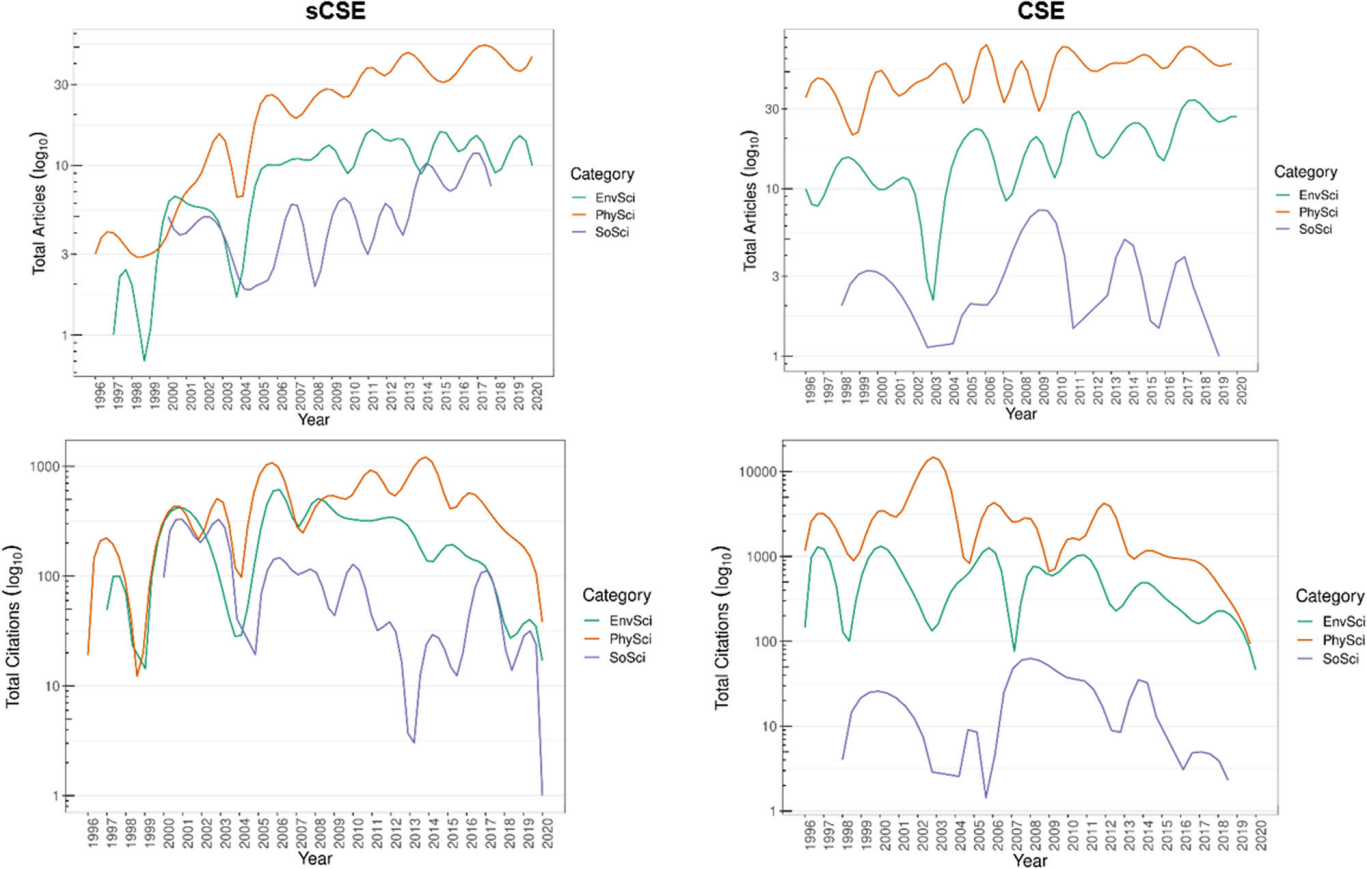
Total articles and citations by category 1996–2020. Left column: sCSE; right column: CSE. Source: the authors based on (Fundación Alejandro Ángel Escobar, 2007, 2020) and Scopus (2020b).

Table 1 presents the bibliometric descriptives of both sCSE and CSE. Figure 3 displays both total output and citations 1996–2020 by category for both sCSE and CSE. *Physics and natural sciences* (PhySci) was the category with the most profiles found in Scopus and the highest number of researchers involved for both sCSE and CSE. It was also the category with the highest annual growth and researchers per publication for the sCSE. In contrast, the highest annual growth for the CSE was for *Environmental Sciences and Sustainable Development* (EnvSci). EnvSci was the category with the highest citation per article for the sCSE, while for the CSE it was PhySci. In sum, all categories in both sCSE and CSE showed positive annual growth, except for *Social Sciences and Humanities* (SoSci).

**Table 1:** sCSE and CSE Scopus profiles and bibliometric descriptive by category 1996–2020.

sCSE							
Category	Scopus profiles	Documents	Articles	Annual growth %	Researchers	Citation per articles	Most relevant periodical
PhySci	18	572	492	12.27	1001	17.12	*Optics Communications*
EnvSci	7	184	177	11.03	266	21.01	*Brittonia*
SoSci	10	69	47	−7.73	53	15.12	*Revista de Economia Institucional*
Total	35	825	716	5.19	1320	17.75	

CSE							
Category	Scopus profiles	Documents	Articles	Annual growth %	Researchers	Citation per articles	Most relevant periodical
PhySci	17	1080	914	1.47	1538	58.73	*Physical Review A*
EnvSci	11	388	348	5.79	549	27.68	*Chemosphere*
SoSci	7	34	28	−3.25	20	3.71	*Revista de Estudios Sociales*
Total	35	1502	1290	1.34	2107	30.04	

Source: (Fundación Alejandro Ángel Escobar, 2007, 2020; Aria and Cuccurullo, 2017; Scopus, 2020a; Minciencias, 2021). Note: EnvSci: *Environmental Sciences and Sustainable Development*; SoSci: *Social Sciences and Humanities*; PhySci: *Physics and Natural Sciences*.

In the sCSE, the most relevant periodical (most frequent) for SoSci was *Revista De Economia Institucional* (Colombia—U. Externado), for EnvSci *Brittonia* (USA—Springer), and for PhySci *Optics Communications* (Netherlands—Elsevier). On the other hand, for the CSE, the most relevant periodicals for SoSci was *Revista de Estudios Sociales* (Colombia—U. Los Andes), for EnvSci *Chemosphere* (UK—Elsevier), and for PhySci *Physical Review A* (US—American Physical Society).

As will be presented in Table 2, displaying individual researcher level data such as full name or affiliation is a common feature of scientific elite studies—even in non-related studies showing highly cited articles or researchers in a given region or institution (Cortés-Sánchez, 2019; Cortés 2021a; Cortés et al., 2021b, 2021a). For instance, Ioannidis et al. (2016) and Li et al. (2020) made publicly available supplementary information on the citation and publication records of more than 84,000+ researchers and Nobel laureates 1900–2016 with the name and even the publication record at the researcher level. In our case, first, the sCSE and CSE are publicly announced and archived on the FAAE website. Second, researcher CvLACs are public—if approved by the researcher—and some researchers even add their Scopus or Publons (WoS) profile link showing their full name, affiliation, country, published documents and citation record. This predilection for the public or private use of a quantitative approach for evaluating every dimension of the higher education system is still, in part, a solid legacy from the ‘New Public Management’ of the 1980s (Gingras, 2014).

**Table 2:** Composite citation indicator (*C*) for both the CSE and sCSE.




Source: the authors based on (Scopus, 2020a, 2021; Ioannidis et al., 2016). Note: *NC* total citations, *H* H index, *Hm* Schreiber Hm index, *NS* total citations for articles of which the scientist is single author, *NSF* total citations for articles of which the scientist is single or first author, *NSFL* total citations for articles of which the scientist is single, first, or last author; *C*: composite citation indicator.

## Methods

We implemented the following methods relative to each RQ:

RQ_1_: a descriptive-longitudinal analysis of total output and citation per category, and the citations per article signalling the period before and after receipt of a special mention. As an exploratory observation, we focused on the top-three most prolific researchers for both the sCSE and CSE.

RQ_2_: we replicated the composite citation indicator (*C*) by Ioannidis et al. (2016). *C* provides a framework for the comparison of individual researchers across different fields. In the cited study, Ioannidis et al. analysed researchers from physics, mathematics or computer science to health sciences and social sciences. Recent studies have used this technique in multidisciplinary contexts, such as the publishing output of COVID-19 across 174 research sub-fields (Ioannidis et al., 2021).

*C* considers multiple indicators for measuring bulk impact (number of citations and *h* index), normalised coauthorship (*hm* index), and author order (total citations to articles as single; single or first; or single, first, or last author). Researcher order signals the role of each author in the development of the manuscript. In biological and physical sciences, for example, the first author is an early-career researcher carrying out the work, while the last and middle author(s) is a mentor figure or principal investigator, and researcher technician with a more specialised role, respectively (Sekara et al., 2018).

The purpose of including multiple and non-redundant metrics is to provide a comprehensive outlook of output, coauthorship, and impact at the individual level. For instance, Ioannidis et al. (2016) found a strong correlation between *h* index and total citations in a sample of 84,000+ researchers. However, there was no correlation between total citations and *hm* index, and a negative correlation between total citations and total citations to articles as single; single or first; or single, first, or last author, indicating that highly cited researchers have published few articles or no single article as single, first, or last researchers.

Among the first aspect of *C* are total citations and *h* index (Hirsch, 2005). The *h* index is defined as follows: for a set of articles *N* of an author and defining *c*_*i*_ as the number of citations corresponding to an article *i* then ordering the set of articles in decreasing order according to the number of citations. The equation for this is:

$$h\,{\mathrm{index}} = {{{\mathrm{max}}}}\,\left\{ {{{i}} \in {{N}}:{{c}}_{{i}} \ge {{i}}} \right\}$$

Source: Hirsch (2005)

The second aspect is the *hm* index (i.e., an h index adjustment for coauthored articles) (Schreiber 2008b, 2008a). For a set of articles *N* with *c*_*i*_ the number of citations for the article *i* and *a*_*i*_ the number of corresponding researchers, the cumulative sum of the inverse of the number of researchers is proposed as the effective rank ${{r}}_{{{{\mathrm{eff}}}}} = \Sigma ^i\frac{1}{{{{a}}_{{i}}}}$. After ordering the set of articles in decreasing order according to the number of citations, the *hm* index can be defined as:

$$hm\,{\mathrm{index}} = \max \left\{ {{{r}}_{{{{\mathrm{eff}}}}} \in {{N}}:{{c}}_{{i}} \ge {{r}}_{{{{\mathrm{eff}}}}}} \right\}$$

Source: Schreiber (2008b, 2008a).

And in the third aspect are the number of citations as a single author; a single or first author; and a single, first, or last author. Finally, their composite is calculated as the sum of the 0–1 log-transformed normalisation of the previous indices. In sum, *C* brings a more nuanced perspective of an author’s impact by including total impact, coauthorship adjustment, and author order as proxy for the leading role (or absence thereof).

## Results

**Fig. 4:**
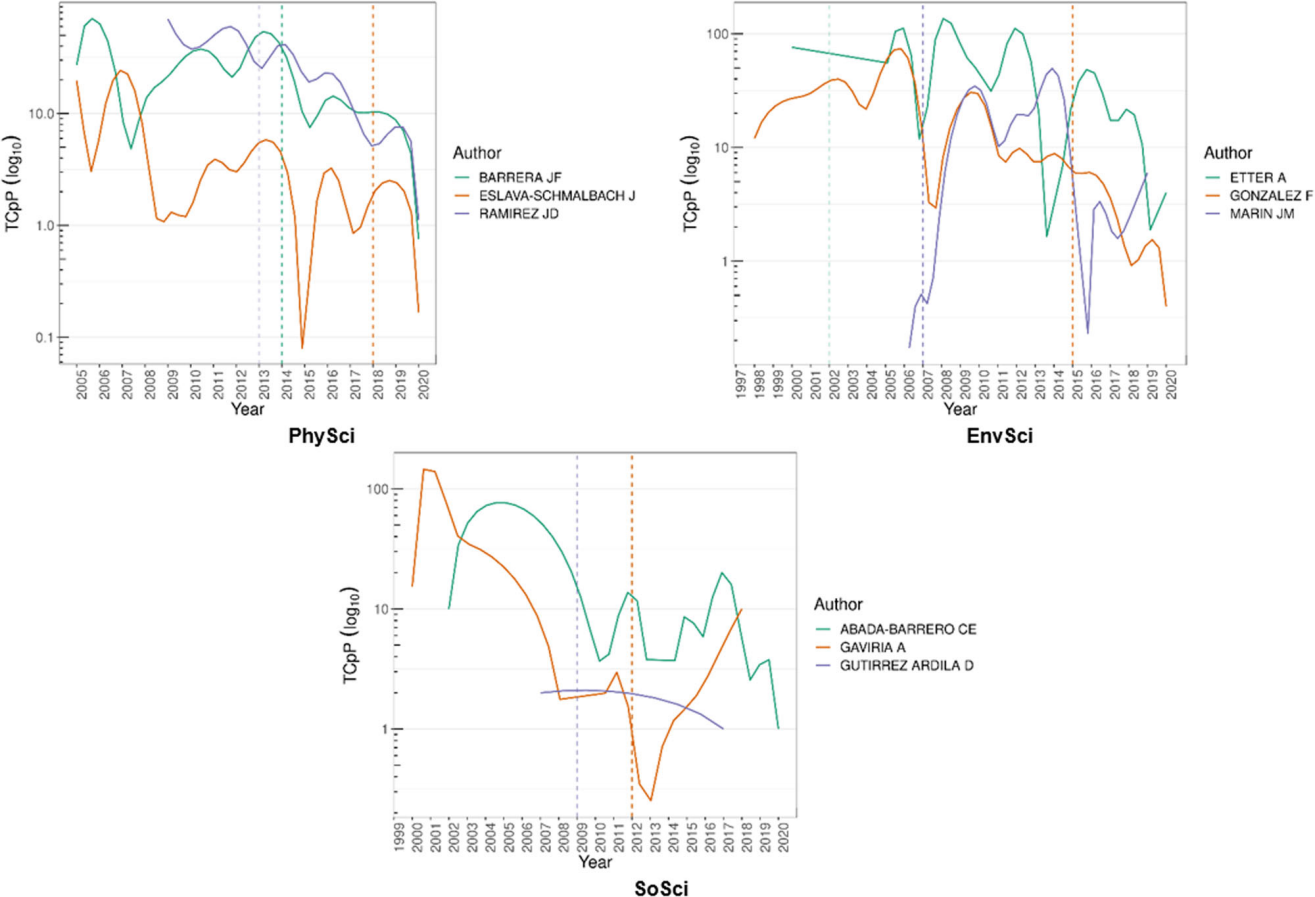
Citations per article by category of top-three most cited researchers for sCSE. Source: the authors based on AAEF (Fundación Alejandro Ángel Escobar, 2007, 2020) and Scopus (2020b). Note: the dashed line points to the year each researcher was awarded the AAEP special mention; TCpP: citations per articles.

**Fig. 5:**
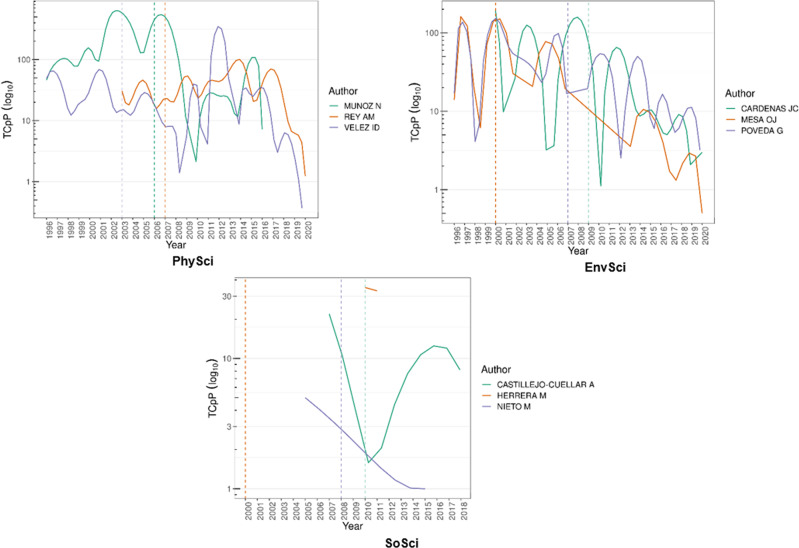
Citations per article by category of top-three most cited researchers for CSE. Source: the authors based on AAEF (Fundación Alejandro Ángel Escobar, 2007, 2020) and Scopus (2020b). Note: the dashed line points to the year each researcher was awarded the AAEP special mention; TCpP: citations per articles.

There is a significant presence of sCSE degrees from and affiliations with prestigious local and global universities. Around 75% of the sCSE have a degree either from a Colombian (~32%) or USA-European (~43%) institution. sCSE affiliations are private, such as Universidad de Los Andes, and public, such as Universidad Nacional de Colombia (Minciencias, 2021). Both are the most prestigious local institutions in each sector—if prestige is at least partially reflected in rankings (Quacquarelli Symonds, 2020). Figures 4 and 5 show the citations per article by category for both sCSE and CSE. The dashed line marks the year each researcher was awarded the AAEP or special mention.

In sCSE-PhySci, John Fredy Barrera (2014) is the most productive researcher with 108 published articles. Barrera is a physicist at Universidad de Antioquia (Colombia). The highest peak of citations per article for this researcher occurred in 2006. The special mention was awarded in 2014 after three major peaks. The second most productive researcher in this category is Javier Eslava Schmalbach, a physician at Universidad Nacional de Colombia, with 89 published articles from 2005 to 2020. For this researcher, a significant and unique peak of citations per article was in 2007. The third most productive researcher is Juan David Ramirez (2013), with 86 published articles. He is a microbiologist at Universidad del Rosario (Colombia). In his case, the number of citations per article showed a major peak in 2009, followed by a downward trend and some smaller peaks thereafter. The special mention was awarded between his second and third peaks. Regarding the CSE-PhySci, the most productive researcher is Nubia Muñoz (2006—physician, emeritus professor at Cancerology National Institute, Colombia) with 224 articles published 1996–2016, followed by Luís Fernando García (2000—physician at U. Antioquia, Colombia) with 152 1996–2020 and Ana María Rey (2007—physicists at U. Colorado, Boulder) with 150 2003–2020. In contrast with the previous sCSE findings, two out of three researchers show multiple peaks after receiving the award. The number of citations per article for Ana María Rey and Iván Darío Vélez (2003—physician at U. Antioquia, Colombia) show their highest peak after receiving the AAEP, while for Nubia Muñoz the highest peak occurred before receiving the award.

In sCSE-EnvSci, the most productive researcher is Favio Gonzales (2015), with 69 articles published. He is a biologist at Universidad Nacional de Colombia and had two major peaks in 2006 and 2010. The special mention was given after those peaks with no subsequent peaks. Juan Marín (2007), in second place, has published 45 articles. He is a chemical engineer at Universidad de Antioquia and had two major citation peaks per article in 2010 and 2014. He was awarded the special mention before his two peaks. The third most productive researcher is Andres Etter (2002), with 45 published articles. He is a biologist at Pontificia Universidad Javeriana (Colombia) and was awarded the special mention before his three significant peaks. Regarding the CSE-EnvSci, the most productive researcher is Jesús Olivero Verbel (2021—chemist at U. Cartagena, Colombia) with 129 articles published 1998–2020, followed by Germán Poveda (2007—engineering at U. Nacional, Colombia) with 123 1996–2020 and Juan Camilo Cárdenas (2009—economist at U. Los Andes, Colombia) with 40 between 2000–2020. For Juan Camilo Cárdenas, Germán Poveda and Óscar Mesa (2000—engineering at U. Nacional, Colombia) their highest citation per article peaks occurred before the AAEP.

For SoSci, the most productive researcher is Cesar Abadía (2012), a medical anthropologist at the University of Connecticut (USA). Abadia has 18 published articles from 2002 to 2020, with a significant peak in 2006. He was awarded the special mention in 2012 (along with Alejandro Gaviria, they both share the same dotted-orange line), several years after his most significant peak. The second most productive researcher is Alejandro Gaviria (2012), with 17 published articles and a single peak in 2001. Gaviria was the president of Universidad de Los Andes until 2021. In third place is Daniel Gutierrez (2009), a historian with 12 articles at Universidad Externado (Colombia). He has had no major peaks in his career. Regarding the CSE-SoSci, the most productive researcher is Sergio Andrés Mejía (2016—historian at U. Nacional, Colombia) with ten articles published 2007–2017, followed by Alejandro Castillejo (2010—anthropologist at U. Los Andes, Colombia) with nine 2007–2019 and Carl Henrik Langebaek (2009—anthropologist at U. Los Andes) with four 2004–2018. The single researcher with the highest citations per article after receiving the AAEP was Alejandro Castillejo, while Mauricio Nieto (2008—historian at U. Los Andes, Colombia) and Martha Herrera (2000—historian U. Los Andes, Colombia) display a decreasing trend; Herrera’s record of indexed publications is an exception as best.

In sum, the special mention in PhySci was awarded after the researchers’ major peaks in citations per article—a similar trend to that found in SoSci. In EnvSci, the special mention came before significant peaks. We can thus deduce that the special mention produced no overall push effect. Indeed, six out of nine researchers received their special mention after multiple peaks, i.e., at the tail-end of a seasoned career. These findings reflect those of the CSE, indicating that no consistent evidence points to a push effect after receiving the AAEP.

**Fig. 6:**
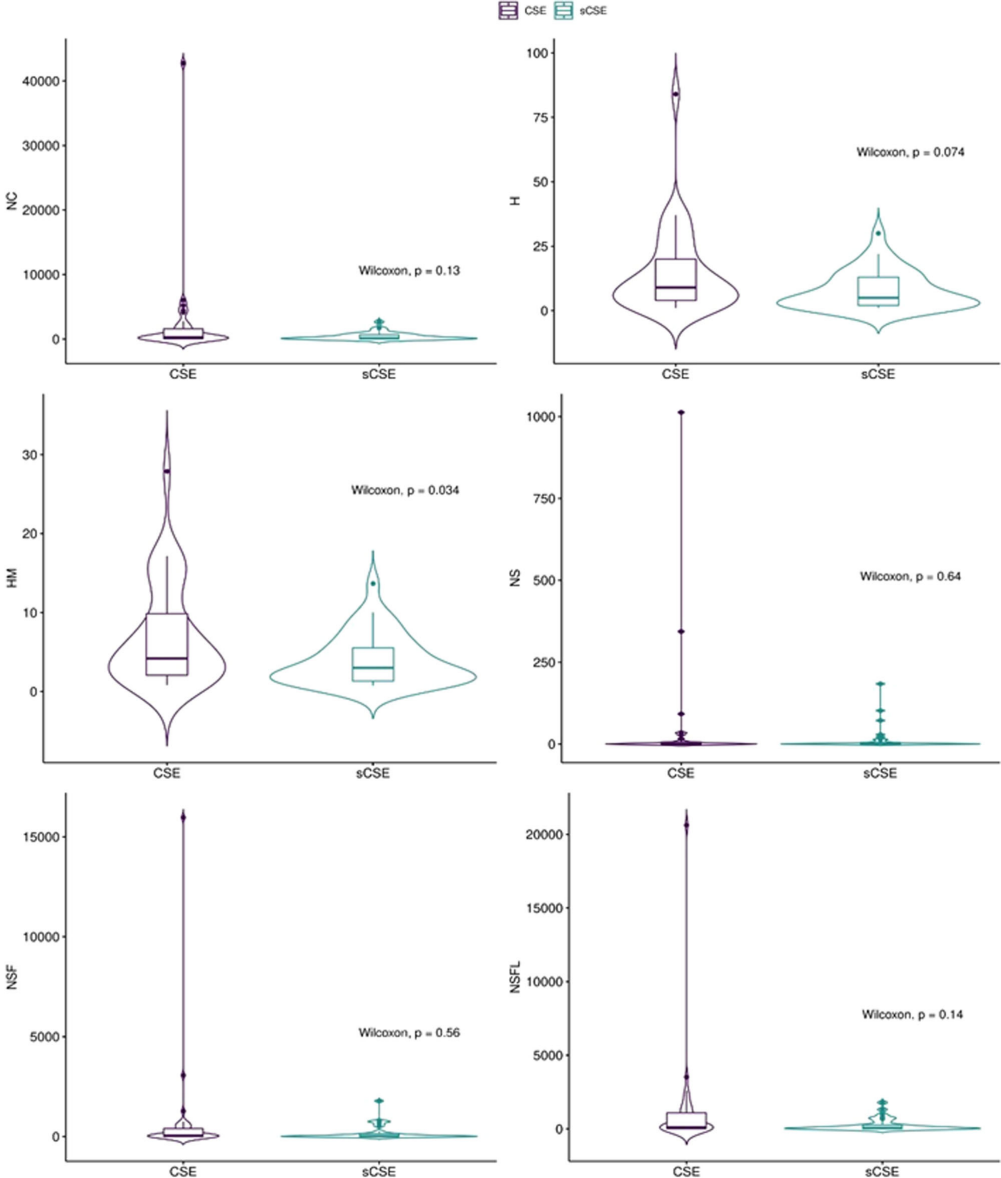
Box-violin plots according to group: CSE and sCSE and bulk impact and authorship order adjusted impact indices. Source: the authors based on (Scopus, 2020a; Ioannidis et al., 2016). Note: NC: total citations; H: H index; Hm: Schreiber Hm index; NS: total citations for articles of which the scientist is the single author; NSF: total citations for articles of which the scientist is the single or the first author; NSFL: total citations for articles of which the scientist is the single, first, or last author.

Table 2 presents the 70 profiles according to *C*, from higher (top) to lower (bottom) scores as listed in the last column. *C* colour-coding goes from dark (higher) to light (lower) green. The first column lists the group: sCSE (light-orange) or CSE (darker-orange). The second column lists the AAEP researcher category: PhySci, EnvSci, and SoSci. The third column lists the re-classification based on the researcher according to the ASCJ subject. The fourth column lists the researcher’s name. Columns five to twelve list the indicators, namely NC: total citations; H: H index; Hm: Schreiber Hm index; NS: total citations for articles of which the scientist is a single author; NSF: total citations for articles of which the scientist is single or first author; NSFL: total citations for articles of which the scientist is single, first, or last author. Indicator colour-coding goes from dark (higher) to light (lower) green. For clarity, Fig. 6 displays the violin-box plot for each indicator by group for a clearer distribution view. Finally, horizontal lines divide the table into quartiles.

There is a mixed composition in all quartiles, meaning that both sCSE and CSE were interleaved throughout the list. In other words, the CSE did not out-perform the sCSE in the 50% superior *C*. Among the CSE there are truly exceptional world-class researchers such as Nubia Muñoz (PhySci), renowned for her work on human papillomavirus, or Ana María Rey (PhySci) for her work on the interface between atomic, molecular and optical physics. Both appear among the 4th quartile group. Appearing in the same quartile are sCSE researchers such as Alejandro Gaviria Uribe (SoSci) and John Fredy Barrera Ramírez (PhySci).

Beginning with the arts and humanities’ *C*, this field was led by sCSE Juan Manuel Toro Soto, a psychologist at Pompeu Fabra University (Spain); followed by Victor Manuel Uribe Urán, a professor of history and law at Florida International University, USA; and Astrid Ulloa, an anthropologist at Universidad Nacional de Colombia. In the ASCJ subject area of biochemistry, genetics and molecular biology, CSE researchers ranked in first and second place: Marlene Jiménez Del Río, a bacteriologist at Universidad de Antioquia; followed by Carlos Alberto Vélez Pardo, a microbiologist at Universidad de Antioquia. In third place was sCSE Silvia Blair Trujillo, a physician at Universidad de Antioquia. Regarding agricultural and biological sciences—as in the arts and humanities—sCSE took the lead. In first place was Favio Antonio González Garavito, a biologist at Universidad Nacional de Colombia; followed by Juan David Ramírez González; and Andrés Etter Rothlisberger. The sole CSE presence in the up-rankings was Juliana Jaramillo Salazar, an anthropologist at University of Hanover, Germany. In medicine, two CSE researchers led the subject field: Nubia Muñoz, a physician who graduated from Universidad del Valle (Colombia); and Iván Darío Vélez Bernal, a physician who graduated from Universidad de Antioquia. The first sCSE appearance was Javier Hernando Eslava Schmalbach.

## Discussion

The sCSE do not have a high publication profile in internationally recognised bibliographic databases since only 31% have published at least two articles indexed in Scopus. The CSE also shares this feature: only ~47% of the complete list have at least two documents indexed in Scopus (Cortés and Andrade, 2022). This reflects the AAEP’s inclusiveness: accepting not only research articles indexed in international indexing systems but also M.Sc. theses; NGO technical reports; local research problems-motivations; and other disciplinary publishing practices and traditions (Larivière et al., 2006; Lisée et al., 2008; Confraria and Godinho, 2015; Confraria et al., 2017; Klavans and Boyack, 2017; Kulczycki et al., 2018; Tollefson, 2018).

Whereas in PhySci and EnvSci both sCSE and CSE found their most relevant periodicals in international (English-language) periodicals, in SoSci both elites found local (Spanish-language) periodicals edited and published by Colombian universities. Such frequency of local and international periodicals is consistent with disciplinary focus, publishing and citation practices—in a word: tradition. For instance, STEM disciplines publish in international journals or conference proceedings published in English, while research in social science and humanities is usually published in books (chapters) and focus on local problems (Larivière et al., 2006; Lisée et al., 2008; Kulczycki et al., 2018; Tollefson, 2018).

The sex disparity in the sCSE is also present in Nobelists—and the scientific workforce in general. In the sCSE, a mere ~24% were female researchers. Among the Nobel laureates, however, the female/male ratio showed a more radical disparity. Despite some ‘progress’—namely that ten women received the Nobel Prize in the last 15: the same number during the first century of the Nobel’s history—just ~2% of female researchers have been honoured with the award (Lunnemann et al., 2019). This is yet more evidence of sex disparity in the sciences, despite both female and male researchers publishing at a comparable annual rate and having a similar impact. For example, out of 16,700+ researchers in Colombia, just ~38% were female (Minciencias, 2019). UNESCO estimates a similar percentage worldwide: ~29% (UNESCO UIS, 2019). Huang et al. (2020) argue that career lengths and dropout rates explain the productive and impact differences between male and female scientists, impacting female researchers seeking to develop their full-potential and acquire senior research positions that would produce more visibility among the scientific elite.

Most of the sCSE have under/graduate studies from Colombia and the US (~57%), with top-tier local (e.g., Nacional and Los Andes) and global (e.g., Harvard, Wisconsin) universities being the most frequent. Among the sCSE such a prestigious academic background is acknowledged to be crucial for more rapid dissemination of ideas than work of a comparable quality from less-reputable institutions (Morgan et al., 2018). This increases institutional inequality in disseminating and circulating knowledge, inducing a *prestige bias* that generates a specific institution’s cumulative advantage (Merton, 1968; Henrich and Gil-White, 2001; Jiménez and Mesoudi, 2019). Institutional *pedigree* can thus be seen as a significant factor in bringing AAEP participants local/international recognition and subsequent admission to the sCSE.

The special mention and AAEP cases examined produced no overall push effect in citation per article for either the sCSE or the CSE; recognition came after a career marked by multiple citation per article peaks—despite some cases in which researchers experienced their highest citation per article peak after receiving the AAEP such as Rey or Vélez in PhySci or Castillejo in SoSci. In contrast, in-progress findings show disparate citations per article trends for the CSE (Cortés and Andrade, 2022), i.e., CSE researchers received the AAEP a number of years before their first peak, some at their peak, and others after their most significant peaks. Research shows that Nobel laureates receive the award at their career peak, followed by a brief *halo effect* (Thorndike, 1920; Gingras and Wallace, 2010). Thus, citation per article trends post-local award/recognition is different to that of Nobel Prize winners. By contrast, the sCSE pattern is comparable to that of the *computer science elites* (i.e., Turing Award) where the average researcher’s age—signifying a longer career with impact-output peaks—has been progressively increasing (Jin et al., 2021). The before/after pushing effect of receiving the special mention analysed here consisted only in an exploratory observation considering the discussion focused on the top-three most prolific researchers per category. Further studies on impact/citation effects after receiving an award could use more robust methodologies, such as regression discontinuity or structural variation analysis (Hou et al., 2021).

On the other hand, there is no clear-cut division between sCSE and CSE. sCSE appeared among the 4th and 3rd quartile researchers, while CSE appeared among the 2nd and 1st quartiles. This was also visible when applying *C* in an attempt to compare the CSE and Nobel laureates (Cortés and Andrade, 2022), an observation also made by Ioannidis et al. (2016). This contrasts with Garfield’s assertion—later contested by Gingras and Wallace (2010)—that it is no longer reliable to identify and separate a scientific elite from other researchers with similar or higher impact in their respective disciplines (Garfield, 1986).

## Conclusion

This study aimed to examine the bibliometric features of individual researcher profiles of the sCSE before and after receiving the recognition and to compare the research impact of that group with that of the CSE via a composite citation indicator. Therefore, contributing to the nascent literature on scientific (semi-)elites in developing countries. Instead of solely considering the work submitted annually to the AAEP and subsequently awarded or given a special mention, the AAEF acknowledges the academic careers of both sCSE and CSE. It also offers a more comprehensive research evaluation by funding and awarding researchers based on the disciplinary incidence and leading roles in a given discipline. This method of adjudication and award places the profile of local researchers—either members or semi-members of an elite—in a more inclusive and heterogeneous framework than that of the scientific elites in higher-income countries (e.g., Clarivate’s Highly Cited Researchers).

The following conclusions can be drawn from our study. First, the broader inclusion of the AAE regarding the type of work and education degree of the representative researcher and of different disciplinary traditions enables the participation of junior and senior researchers, the paucity of which is noticeable from the lower record of documents indexed in international systems such as Scopus. Second, the sex disparity in the sCSE reflects both a national and worldwide trend. Third, there was a significant presence of sCSE degrees from and affiliations with prestigious local and global universities, which could be a factor in their inclusion in the sCSE and the more rapid dissemination of ideas. Fourth, we found no overall *halo effect* in citations per article after receiving the special mention. This supports the ongoing findings for the CSE, but contrasts with the *halo effect* that surrounds the Nobel laureates after receiving the award at their citation per article peak. Finally, there was no clear-cut division between the *C* of sCSE and CSE, a finding that needs to be considered when studying scientific (semi-)elites in a context outside higher-income countries.

Our study has several limitations. On the one hand, our approach was limited to one developing country and one bibliographic database only. Further research could examine other awards in developing countries (e.g., the *Prêmio Almirante Álavaro Alberto*, Brazil; or the *Highest Science and Technology Award*, China) using multiple or integrated bibliographic databases (e.g., WoS, Dimensions, Google Scholar, among others). Also, science mapping techniques could also be used to ascertain the structural features of the sCSE, CSE, and other elites. Finally, the inclusion of *altmetrics* could look at the disciplinary incidence of scientific elites in public debates in social media.

## Data Availability

Scopus bibliographic data are not available due to commercial restriction but are available from the corresponding author on reasonable request.
